# Genetic diversity of a large set of horse breeds raised in France assessed by microsatellite polymorphism

**DOI:** 10.1186/1297-9686-41-5

**Published:** 2009-01-05

**Authors:** Grégoire Leroy, Lucille Callède, Etienne Verrier, Jean-Claude Mériaux, Anne Ricard, Coralie Danchin-Burge, Xavier Rognon

**Affiliations:** 1 AgroParisTech, UMR1236 Génétique et Diversité Animales, 16 rue Claude Bernard F-75321 Paris, France; 2 INRA, UMR1236 Génétique et Diversité Animales, 78352 Jouy-en-Josas, France; 3 LABOGENA, F-78352 Jouy-en-Josas, France; 4 INRA, UR631 Station d'amélioration génétique des animaux, BP 52627, 31326 Castanet-Tolosan, France

## Abstract

The genetic diversity and structure of horses raised in France were investigated using 11 microsatellite markers and 1679 animals belonging to 34 breeds. Between-breed differences explained about ten per cent of the total genetic diversity (Fst = 0.099). Values of expected heterozygosity ranged from 0.43 to 0.79 depending on the breed. According to genetic relationships, multivariate and structure analyses, breeds could be classified into four genetic differentiated groups: warm-blooded, draught, Nordic and pony breeds. Using complementary maximisation of diversity and aggregate diversity approaches, we conclude that particular efforts should be made to conserve five local breeds, namely the Boulonnais, Landais, Merens, Poitevin and Pottok breeds.

## Introduction

During the twentieth century, horse breeding has undergone large changes in Europe. Previously considered as an agricultural, industrial and war tool, horse is now essentially bred for hobby riding. Draught horses, in particular, have been less and less used as utility horses, and many draught breeds have undergone a dramatic decrease in population size: according to the Haras Nationaux, out of the nine French draught breeds, six have annual births below 1000. Measures for *in situ *conservation have been applied in France for several years but such measures are in general expensive. Therefore, it would be useful to identify priorities among conservation purposes and this requires characterising diversity and genetic relations between breeds [1].

During the last fifteen years, microsatellite markers have frequently been used to evaluate genetic distances and to characterise local breeds, [2-10]. Some methods have recently been developed to evaluate the genetic contribution of populations to within-breed and between-breed diversities [11,12].

With about 800 000 animals belonging to 50 different breeds (source: Haras Nationaux), France shows a large diversity of horse populations. Among these breeds, 21 have a French origin or have been bred in France for at least a century. According to the FAO, at least 15 populations have disappeared during the last 50 years, and eight indigenous breeds are still considered as endangered or endangered-maintained. Among those breeds, the majority are draught breeds, namely the Ardennais, Auxois, Boulonnais, Poitevin and Trait du Nord breeds, the other ones being the Merens warm-blooded breed and the Landais and Pottock pony breeds. Information on the genetic diversity of French endangered breeds could help breeders and providers, decide where they should place more emphasis.

In the present study, we first analysed the genetic diversity of 39 horse populations reared in France: within-breed diversity, breed relationship and population structure were investigated, using microsatellite data. Then, we focussed on 19 breeds of French origin or having been raised in France for at least a century, and evaluated the conservation priorities between these populations, using different approaches to evaluate within, between and total diversity.

## Methods

### Populations sampled and microsatellite analysis

French nomenclature divides horse breeds into three groups: warm-blooded, draught horses and ponies. In this study, 39 populations were considered (Table 1). These 39 populations comprised 31 recognised breeds (including 13 warm-blooded breeds, nine draught breeds, and nine pony breeds), the primitive Przewalski horse (used as an outgroup), and seven populations originating from the splitting of two recognised breeds, namely the Anglo-Arab (AA) and Selle Français (SF) breeds (divided into four and three groups, respectively). The 2005 studbook rules define those groups according to the proportion of foreign genes that can be found from genealogical analysis: AA6 and AA9 are considered as pure AA, whereas AA5 and AA10 can have ancestors from another origin, the proportion of Arab origin being higher for AA5 and AA6 than the others. SF8 has a large proportion of PS origin and can therefore be used to produce AA, SFA97 constitutes a group closed to direct foreign influences, whereas SFB98 individuals can have a parent from another breed (under some conditions).

**Table 1:** Basic information on the 39 populations studied

Population code	Breed	Type^a^	Country^b^	Nb of foals registered in 2005	Sample size^c^
**AA10**	Anglo-Arab	W	France	282	50 (13)

**AA5**				781	50 (11)

**AA6**				244	50 (15)

**AA9**				252	50 (11)

**APPAL**	Appaloosa	W	USA	84	29

**AB**	Arab-Barb	W	Morocco	71	38

**AR**	Arab	W	France	1267	50

**ARD**	Ardennais	D	France	645	50

**AUX**	Auxois	D	France	130	35

**BA**	Barb	W	Morocco	99	24

**BOUL**	Boulonnais	D	France	290	49

**BR**	Breton	D	France	3548	50

**CAM**	Camargue	W	France	468	37

**CO**	Connemara Pony	P	Ireland	456	49

**COBND**	Cob Normand	D	France	495	50

**COMT**	Comtois	D	France	4173	50

**FJ**	Fjord	P	Norway	237	33

**FRI**	Friesian	W	The Netherlands	53	37

**HAF**	Haflinger	P	Austria	344	32

**IS**	Iceland Pony	W	Iceland	96	48

**LAND**	Landais	P	France	31	27

**LUS**	Lusitanian	W	Portugal	312	50

**MER**	Merens	W	France	443	32

**NF**	New Forrest Pony	P	UK	119	45

**PER**	Percheron	D	France	1309	50

**PFS**	Poney français de selle	P	France	1069	50

**POIT**	Poitevin	D	France	90	35

**POT**	Pottok	P	France	170	50

**PRE**	Pure Spanish Horse	W	Spain	146	50

**PRW**	Przewalsky horse	Pr	Mongolia	-	26

**PS**	Pur Sang (Thoroughbred)	W	France	4822	50

**QH**	Quaterhorse	W	USA	162	41

**SF8**	Selle Français	W	France	732	50 (17)

**SFA97**				5729	50 (20)

**SFB98**				895	50 (13)

**SHE**	Shetland Pony	P	UK	402	50

**TDN**	Trait du Nord	D	France	96	23

**TF**	Trotteur Français	W	France	10348	50

**WAB**	Welsh Pony	P	UK	142	39

^a^W = warm-blooded horse, D = draught horse, P = pony, Pr = primitive horse

^b^France = breeds of French origin or raised in France for at least 100 years; other countries = country of origin for breeds raised in France for less than 100 years

^c^In brackets, number of individuals of each AA and SF subpopulation used when aggregating the four and three subpopulations, respectively

For each of the 39 populations, 23 to 50 animals born between 1996 and 2005, were sampled amounting to 1679 animals. Except for the Przewalski horse, where no pedigree data was available, the sampled animals were known to have no common parents. For the conservation approach, the study focussed on 19 populations, either of French origin, or having been bred in France for at least 100 years (PS, AA and AR breeds). In this approach, 50 animals were randomly sampled among the four and three AA and SF subpopulations, respectively, to constitute two populations.

Eleven microsatellite markers were used to perform the analysis (*AHT4, AHT5, ASB2, HMS1, HMS3, HMS6, HMS7, HTG4, HTG6, HTG10, VHL20*), with all but two (*HMS1 *and *HTG6*) being recommended by the International Society of Animal Genetics for parentage testing and used in similar studies (except *HMS1*) [7,9,10]. For the entire sample, amplifications and analyses were performed by the same laboratory, using a capillary sequencer (ABI PRISM 3100 Genetic Analyzer, Applied Biosystems).

### Statistical analysis

Allele frequencies, mean number of alleles *(MNA)*, observed (*Ho*) and non-biased expected heterozygosity (*He*), were calculated using GENETIX [13]. Wright Fis, Fit and Fst coefficients were also computed using the same software. GENEPOP [14] was used to evaluate pairwise genetic differentiation between breeds [15] and departure from Hardy-Weinberg equilibrium, using exact tests and sequential Bonferonni correction [16] on loci. Global tests on Hardy-Weinberg equilibrium were also performed using GENEPOP. Allelic richness was computed using FSTAT [17].

The matrix of Reynolds unweighted distances *D*_*R *_[18] was computed using POPULATION (Olivier Langella; http://bioinformatics.org/~tryphon/populations/). Regarding the D_R _distance, a NeighborNet tree was drawn using SPLITSTREE 4.8 [19]. A factorial correspondence analysis (without the Przewalsky horse) was also performed using GENETIX. Finally, the genetic structure of the populations was assessed using Bayesian clustering methods developed by Pritchard (STRUCTURE, [20]): using a model with admixture and correlated allele frequencies, we made 20 independent runs for each value of the putative number of sub-populations (*K*) between 1 and 22, with a burn-in period of 20 000 followed by 100 000 MCMC repetitions. Pairwise similarities (*G*) between runs were computed using CLUMPP [21].

To evaluate the conservation priorities in a set of populations, taking into account contributions to within-population and between-population genetic diversity, Ollivier and Foulley [12] have proposed the following method. First, the between-breed contribution (*CB*) is evaluated, based on the Weitzman [22] loss *Vk *of diversity when the population *k *is removed from the whole set of breeds (in this study we used *D*_*R *_distance). Then, the within-breed contribution (*CW*) is defined as:

(1) *CW *= 1 - *H(S/k)*/*H(S)*

where *H(S) *is the average internal heterozygosity of the whole set *S *and *H(S/k) *the average internal heterozygosity of the set when *k *is removed. Finally, the aggregate diversity *D *of a population is defined as:

(2) *D *= *F*_*st *_*CB *+ (1 - *F*_*st*_)*CW*.

The cryopreservation potential (*CP*) could be computed as the product between the breed contribution (*CB*) and the probability of extinction (*P*_*ex*_) of the breed, assumed to be directly proportional to the inbreeding rate (*ΔF*). Following Simianer *et al*. [23], *P*_*ex *_can be approximated as

(3) *P*_*ex *_= *c ΔF *= *c*/(2*Ne*) = c (*M *+ *F*)/8 *MF*

where *Ne *is the effective population size, *M *and *F *are the numbers of breeding males and females, respectively, used inside the breed in 2005, and *c *is a constant, to be chosen. Considering that the effective population size of a breed should not be lower than 50 to avoid extinction in the short term [24], we considered that *P*_*ex *_= 1 for *Ne *= 50. Therefore, *c *was set to 100 (see equation 3).

Caballero and Toro [11] have developed a parallel approach. The total diversity *GD*_*T *_can be considered as the exact sum of the gene diversity within population *GD*_*WS *_and the gene diversity between populations *GD*_*BS *_considering the following equations:

(4) *GD*_*T *_= 1 - Σ_*i*_Σ_*j*_*f*_*ij*_/*n*^2^

(5) *GD*_*WS *_= 1 - Σ_*i*_*f*_*ii*_/*n*

(6) *GD*_*BS *_= Σ_*i*_Σ_*j*_*D*_*ij*_/*n*^2^

where *n *is the number of populations, *f*_*ij *_is the average coancestry between populations *I *and *j, and D*_*ij *_is the Nei minimum distance between populations *I *and *j*. The contribution of a population to the diversity is evaluated by computing the loss or gain of diversity *ΔGD *when the population is removed.

The authors have also proposed to evaluate the contributions (*c*_*i*_) of each population, which can maximise the total diversity at the next generation, using the following equation:

(7) *GD*_*TN *_= 1 - Σ_*i *_*c*_*i *_[*f*_*ii *_- Σ_*j *_*D*_*ij *_*c*_*j*_].

The contributions can be computed by maximising *GD*_*TN *_in equation (7), with the following restrictions: for each population *i*, *c*_*i *_≥ 0 and Σ_*i *_*c*_*i *_= 1.

## Results

### Genetic variations

One hundred and nine alleles were found over all populations and all markers. The average number of alleles per locus was 9.8 ranging from seven (locus *HTG4 *and *HMS1*) to 15 (locus *ASB2*). Some rare alleles in the whole data set were found with a high frequency in the PRW population: for instance, with the *HTG6 *loci, the two most frequent alleles in the PRW population (70%) were seldom found in other breeds (less than 1%). Heterozygosities, mean number of alleles (*MNA*) and allelic richness (*AR*) are presented in Table 2. *MNA *and *AR *were highly correlated, (*r *= 0.98, *P *< 0.0001). *He *ranged from 0.43 in the FRI breed to 0.79 in the PFS breed, while Fis per breed ranged from -0.08 (TDN breed) to 0.11 (PRE breed).

**Table 2:** Values for parameters of polymorphism within the 39 populations studied

Population code	*He*	*Ho*	*F*_*is*_	*HWE *deficiency	*MNA*	*AR*
**AA10**	0.71	0.72	-0.01	0	5.45	5.0
**AA5**	0.73	0.71	0.03	0	5.73	5.4
**AA6**	0.73	0.71	0.03	0	5.91	5.3
**AA9**	0.69	0.70	-0.01	0	4.91	4.6
**APPAL**	0.77	0.72	0.06	0	7.55	6.9
**AB**	0.76	0.74	0.03	1	7.00	6.7
**AR**	0.72	0.66	0.08	1	6.09	5.4
**ARD**	0.64	0.62	0.03	0	6.09	5.5
**AUX**	0.65	0.62	0.05	1	6.00	5.5
**BA**	0.74	0.74	0.00	0	7.00	6.8
**BOUL**	0.62	0.60	0.03	1	5.09	4.7
**BR**	0.66	0.67	-0.02	0	6.36	5.8
**CAM**	0.73	0.68	0.07	1	6.36	6.0
**CO**	0.75	0.73	0.03	1	6.64	6.1
**COBND**	0.72	0.73	-0.01	0	6.64	6.1
**COMT**	0.69	0.67	0.03	2	6.00	5.6
**FJ**	0.67	0.69	-0.03	0	6.00	5.6
**FRI**	0.43	0.43	0.00	0	3.45	3.2
**HAF**	0.65	0.62	0.05	0	4.82	4.6
**IS**	0.70	0.68	0.03	1	6.27	5.7
**LAND**	0.75	0.71	0.05	1	6.82	6.6
**LUS**	0.74	0.71	0.04	1	6.27	5.9
**MER**	0.70	0.71	-0.01	0	5.91	5.6
**NF**	0.76	0.74	0.03	1	7.64	6.9
**PER**	0.68	0.69	-0.01	0	6.64	6.0
**PFS**	0.79	0.79	0.00	0	8.09	7.2
**POIT**	0.57	0.58	-0.02	0	4.82	4.4
**POT**	0.77	0.79	-0.03	0	7.82	7.1
**PRE**	0.70	0.62	0.11	1	6.55	5.7
**PRW**	0.59	0.56	0.05	0	3.73	3.7
**PS**	0.69	0.70	-0.01	0	5.00	4.6
**QH**	0.73	0.72	0.01	0	7.00	6.2
**SF8**	0.71	0.73	-0.03	0	5.55	4.9
**SFA97**	0.74	0.73	0.01	0	6.27	5.7
**SFB98**	0.75	0.75	0.00	0	7.00	6.1
**SHE**	0.69	0.65	0.06	0	6.00	5.2
**TDN**	0.64	0.69	-0.08	0	5.36	5.3
**TF**	0.70	0.69	0.01	1	6.27	5.5
**WAB**	0.76	0.74	0.03	0	7.55	7.0

*He *= non biased heterozygosity; *Ho *= observed heterozygosity; *MNA *= mean number of alleles; *AR *= allelic richness; *HWE *deficiency: number of loci deviating from Hardy-Weinberg equilibrium after Bonferroni correction

Some significant heterozygote deficits after corrections were found, for different loci and populations (see Table 2). Only one test exhibited significant excess (AA5 with *HMS1*). Using global tests, five populations (AB, AR, AUX, CAM, PRE) and two markers (*HMS3 *and *HTG10*) showed significant deficit in heterozygotes (*P *< 0.01). Other studies have shown similar results for these two markers [4].

Testing population differentiation, 11 pairs of populations were found non significantly differentiated out of the 741 tests performed: AA5 with AA6, AA9 with AA10, SF8 and PS, PS and SF8, AA10 with SF8 and PS, AB with BA, APPAL with QH, AUX with TDN, SFA97 with SFB98.

The *Fis*, *Fit*, and *Fst *values were 0.019, 0.116 and 0.099, respectively. We found a gene differentiation coefficient *G*_*ST *_[25] of 0.0989.

### Breed relationships and clustering

**Figure 1:**
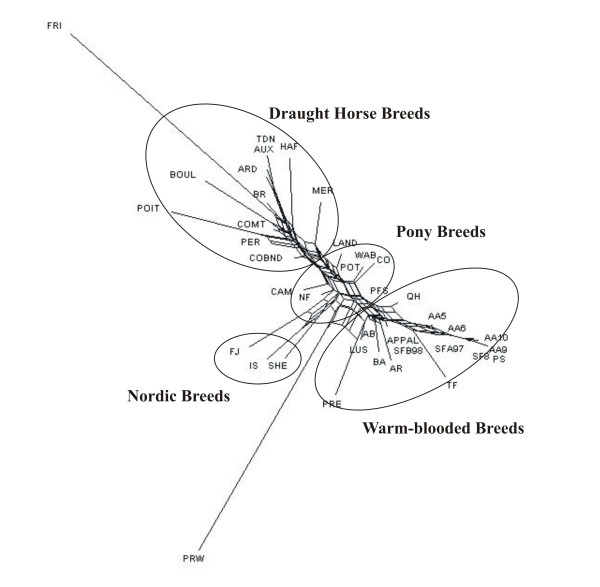
**Neighbour-Net for the 39 horse populations, based on Reynolds D_R _distance**.

The NeighborNet network (Figure 1) clearly separated draught horses (also including MER, HAF breeds) and warm-blooded horses, whereas most pony breeds were placed between these two groups. Nordic (IS, SHE, FJ) breeds formed a separate group. FRI and PRW populations were isolated from the other breeds, the closest groups being draught horses and Nordic breeds, for the FRI breed and PRW population, respectively.

**Figure 2:**
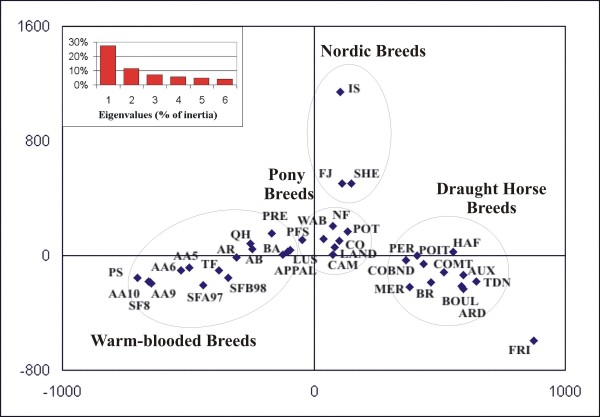
**Correspondence analysis of allele frequencies for 38 of the populations studied (PRW is not included)**. The projection is shown on the first two axes.

In Figure 2, the 38 populations (PRW being excluded) were placed according to the two main axes of the correspondence analysis (accounting for 27.4% and 11.5% of the inertia, respectively). Axis 1 clearly differentiates warm-blooded horses, ponies and draught horses, whereas axis 2 separates Nordic horses (IS, SHE, FJ) from the other ones. The FRI breed seems to be isolated from the other populations, the closest populations being the draught breeds.

Neighbornet and FCA approaches were also used on 34 and 33 breeds, respectively (the four samples of AA breed and three samples of the SF breeds being aggregated into two samples of 50 animals each), showing similar results to previous figures (see Additional files 1 and 2).

**Figure 3:**
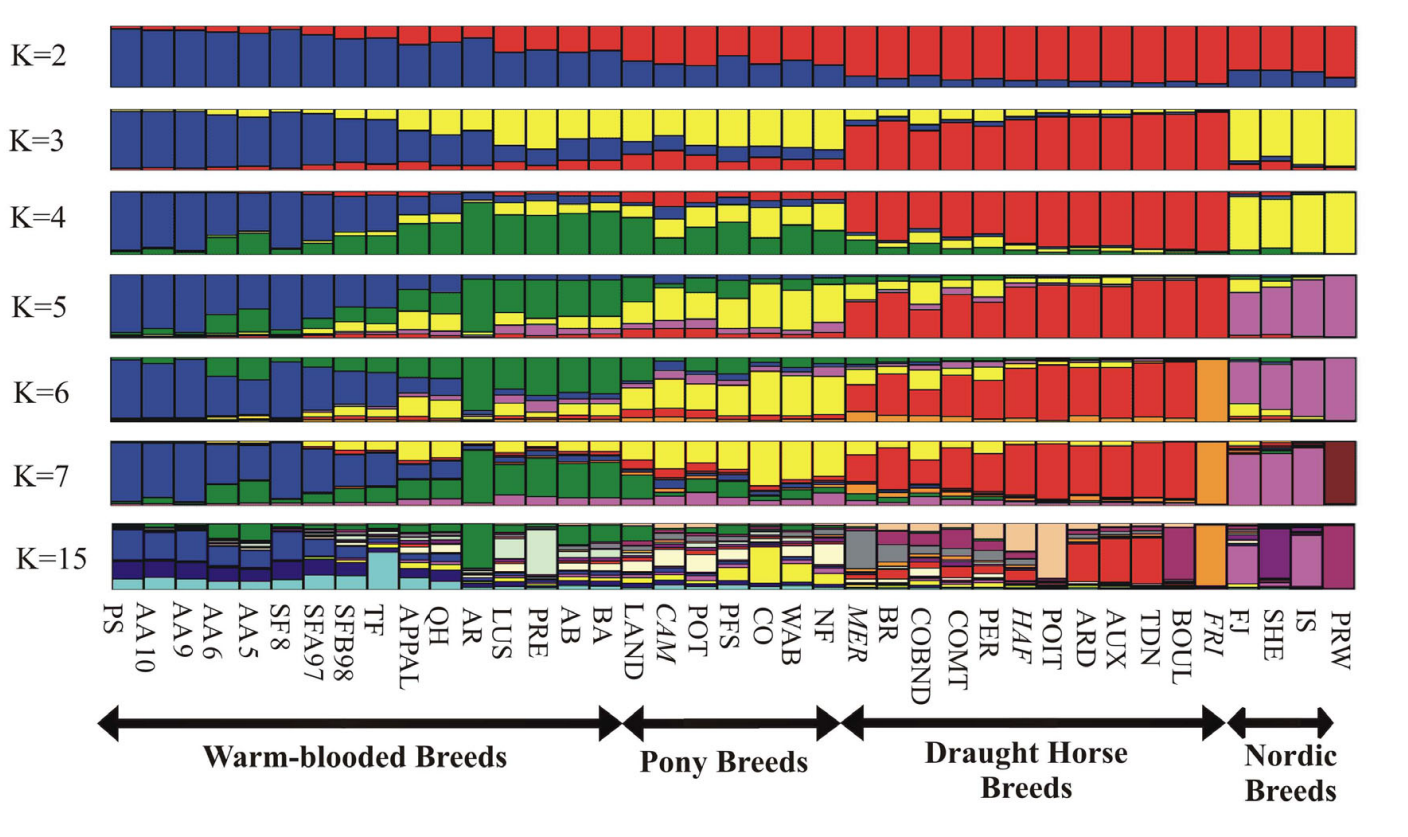
**Cluster assignment of each of the 39 populations to the K cluster**. Among 20 runs, solutions having the most similar pair-wise similarity coefficients are presented here. Breeds not classified in their group according to French nomenclature are in italic.

Breed assignment to clusters provides complementary information on genetic relationships between populations. As *K *increases from 2 to 7, mean similarity coefficients among runs are respectively equal to 0.997, 0.993, 0.993, 0.773, 0.562, and 0.658, respectively. Likelihood increased until *K *reached 15–18 values (see additional file 3), indicating that the most significant subdivisions were obtained for such values. Since mean similarity coefficients were slightly lower for *K *= 16 (0.78) or 17 (0.81) than for *K *= 15 (0.83), the results are shown for this last value. Figure 3 shows the assignment of populations to clusters for each *K*, using runs having the highest pair-wise similarity coefficients.

For *K *= 2, there was a clear separation between draught and warm-blooded horses, with other populations showing intermediate results. When *K *reached 3, Nordic/primitive breeds, ponies, and some warm-blooded horses segregated more or less clearly from the two other clusters. As *K *increases to 4 and 5, the five clusters were constituted of Nordic/primitive breeds, draught horses, ponies, warm-blooded populations close to the AR breed and warm-blooded populations close to the PS breed. Some breeds were shared among the last three clusters, such as LAND between ponies and AR groups, and APPAL among the three clusters. When *K *reached 6, depending on the runs, FRI or PRW populations were alternately isolated, which led to a decrease of similarity across runs and explains the low similarity coefficient (0.562) in comparison with other *K*. When *K *= 7, these two populations were isolated. The different runs highlight some differences among sub-populations of AA and SF breeds, underlining a more important proportion of AR genes in AA6, AA5 and respectively SFA97 and SF98 groups. Some warm-blooded (FRI until *K *= 6, MER) and pony breeds (HAF) were classified with draught horses, while the CAM warm-blooded breed was clustered with ponies. As *K *reached 15, most breeds were shared among different clusters. The ARD, AUX and TDN breed constituted a single cluster while FJ/IS and LUS/PRE constituted two others. In a few cases, a single cluster was essentially associated to a single breed (BOUL, FRI, SHE, PRW).

### Partition of diversity

In the set of the 19 French breeds, we found a gene diversity within population *GD*_*WS *_of 0.685, a gene diversity between populations *GD*_*BS *_of 0.073, and a total gene diversity *GD*_*T *_of 0.758. Table 3 shows between-breed, within-breed, and total contribution/variation of diversity according to Ollivier and Foulley [12] and Caballero and Toro [11] approaches. For within-breed diversity, *CW *and Δ*GD*_*WS *_ranged from -0.48 to 0.50 and from -0.0055 to 0.0069 respectively. In both cases, the POIT breed showed a particularly low within-breed diversity. *CW *and Δ*GD*_*WS *_were negatively correlated (*r *= -0.715, *P *= 0.001). For between-breed diversity, *CB *and Δ*GD*_*BS *_ranged from 0.85 to 12.60 and from -0.0041 to 0.0024, respectively. Here, the POIT breed showed a particularly high contribution to the between-breed diversity. The correlation between *CB *and Δ*GD*_*BS *_was not significant. *D *and Δ*GD*_*T*_, accounting for total diversity, were negatively correlated (*r *= -0.53, *P *< 0.019). They ranged from -0.32 to 1.25 and from -0.0042 to 0.0039, respectively. In both cases, the ARD and PS breeds showed a particularly low and high diversity, respectively.

**Table 3:** Contributions of the different breeds to genetic diversity according to different approaches

Breed code	Nb of breeding animals in 2005		Pr. extinction	Aggregate diversity and cryopreservation potential (Ollivier and Foulley, 2005)				Loss or gain of diversity when a breed is removed and contributions to optimal diversity (Caballero and Toro, 2002)			
			
	Males	Females		*CW*	*CB*	*D*	*CP*	Δ*GD*_*WS*_	Δ*GD*_*BS*_	Δ*GD*_*T*_	*C*_*i*_
**AA**	119	1443	0.11	0.35	0.85	0.39	0.10	-0.0013	-0.0018	-0.0031	0%
**AR**	480	2130	0.03	0.29	10.90	1.25	0.35	-0.0015	-0.0010	-0.0026	0%
**ARD**	187	1417	0.08	-0.48	1.33	-0.32	0.10	0.0031	0.0001	0.0032	0%
**AUX**	24	248	0.57	-0.19	3.14	0.11	1.79	0.0023	-0.0005	0.0018	0%
**BOUL**	58	540	0.24	-0.27	12.35	0.87	2.95	0.0040	-0.0023	0.0018	6%
**BR**	621	6380	0.02	-0.38	5.57	0.16	0.12	0.0016	0.0009	0.0024	0%
**CAM**	118	837	0.12	0.00	7.99	0.73	0.97	-0.0018	0.0013	-0.0006	0%
**COBND**	63	760	0.21	-0.06	2.42	0.16	0.52	-0.0017	0.0019	0.0002	2%
**COMT**	856	7073	0.02	-0.25	3.63	0.11	0.06	0.0000	0.0015	0.0015	0%
**LAND**	22	73	0.74	0.06	3.99	0.41	2.95	-0.0029	0.0016	-0.0014	2%
**MER**	93	1012	0.15	-0.04	10.41	0.91	1.53	0.0000	0.0001	0.0001	0%
**PER**	183	2461	0.07	-0.32	4.60	0.12	0.34	0.0006	0.0014	0.0020	0%
**PFS**	100	949	0.14	0.39	1.93	0.53	0.27	-0.0055	0.0024	-0.0031	70%
**POIT**	39	199	0.38	-0.43	12.60	0.75	4.83	0.0069	-0.0030	0.0039	0%
**POT**	94	910	0.15	0.19	1.33	0.29	0.20	-0.0040	0.0024	-0.0016	5%
**PS**	369	8049	0.04	0.50	6.17	1.02	0.22	-0.0001	-0.0041	-0.0042	1%
**SF**	474	11700	0.03	0.45	1.33	0.53	0.04	-0.0024	-0.0013	-0.0037	15%
**TDN**	16	183	0.85	-0.17	1.93	0.02	1.64	0.0032	-0.0009	0.0022	0%
**TF**	527	15950	0.02	0.36	7.51	1.01	0.18	-0.0002	-0.0029	-0.0032	0%

Sum				0	100	9.054		0	-0.043	0.043	100%

*CW *= contribution to within-breed diversity; *CB *= contribution to between-breed diversity; *D *= aggregate diversity;*CP *= Cryopreservation potential; Δ*GD*_*WS *_= Loss or gain of gene diversity within populations when breed is removed; Δ*GD*_*BS *_= Loss or gain of gene diversity between populations when breed is removed; Δ*GD*_*T *_= Loss or gain of total diversity when the breed is removed; *C*_*i *_= contribution of the breed to optimise *GD*_*T*_

Considering contributions to the between-breed diversity and probabilities of extinction, the BOUL, LAND and POIT breeds showed the highest cryopreservation potentials (2.95, 2.95 and 4.83, respectively).

Contributions of each population for an optimal *GD*_*T *_are given in Table 3: the composite PFS breed should contribute to 70% of the pool, for a total *GD*_*T *_of 0.79. Besides, to maximise the total gene diversity, seven of the 19 breeds should be maintained, namely the BOUL, COBND, LAND, PFS, POT, PS and SF breeds.

## Discussion

### Gene diversity and genetic relations among breeds

Differences between breeds explained 10% of the total genetic variation, which is quite similar to other analyses, where values ranged from 8% to 15% [2-4,9]. According to previous studies using microsatellites, expected heterozygosities ranged from 0.47 for the FRI breed [6] to 0.80 for the Sicilian Indigenous breed [6]. In our study, only one result was found outside this range of values: 0.43 for the FRI breed,* i.e*. close to the value found by Luis *et al*. [6]. Plante *et al*. [9] recently analysed 22 Canadian and Spanish populations. Our estimated values of *He *were slightly lower (0.71 on average vs. 0.75, *P *= 0.048) for the eight breeds shared between their study and the present one. Differences on the within-breed diversity among studies using microsatellites can be explained, on the one hand, by the loci used and, on the other hand, by the populations analysed, incidentally belonging to similar breeds but having different recent histories. In the AR breed, we found a *He *value of (0.72) with a significant deficit of heterozygotes, which can be explained by the fact that this is an international breed in which mating between close relatives is common [26]. Plante *et al*. [9] and Luis *et al*. [6] have found similar results for the same breed, but not Aberle *et al*. [2] who observed a lower heterozygosity (0.57) without a heterozygote deficit. The PER population seemed to have a particularly high genetic diversity in the Plante study (*He *= 0.78), in comparison with the French PER population (*He *= 0.68). Because PER populations have been bred in America since the end of the 19^th ^century, such results should be interpreted bearing in mind that the French PER population has probably suffered from recent bottlenecks due to several modifications of the selection aims.

The three approaches based on genetic relationships (genetic distances, FCA and clustering methods) gave similar results. The populations considered in the present study can be classified into four more or less differentiated clusters: warm-blooded, draught, Nordic and pony breeds. Similar patterns of clustering have been found in other studies [2,3,9,10]. The draught horses constitute a quite homogenous group, including the nine French draught horse breeds and three breeds presently classified as pony (HAF) or warm-blooded (MER and FRI in a lesser extent) breeds. These three breeds were historically used as draught horse breeds and could therefore have been subject to crossbreeding with other draught horse populations in their past history. Pony breeds formed a group in an intermediate position in comparison to the other clusters. It also included the CAM breed, today recognised as a warm-blooded breed, but morphologically considered as a pony [27]. According to our analysis, FRI and PRW populations were found to be genetically isolated, which can be, to some extent, linked to a low genetic variability [28] due to historical bottlenecks within these breeds [2,29]. Moreover, another parameter explaining isolation of the PRW breed is the presence of rare alleles, which was in agreement with other studies [2] and expected for a population considered as a primitive wild horse.

Population differentiation tests and Bayesian approaches indicate clear differences between sub-populations of AA and SF. Such results may be largely explained by differences in the proportion of thoroughbred (PS) origins in the gene pool of these sub-populations. Within the AA breed, AA5 and AA6 populations appeared distinct from AA9 and AA10 populations and close to the PS breed. This was in agreement with the studbook rules: on the basis of pedigree data, AA5, AA6, AA9 and AA10 populations were indeed found to have respectively 94%, 89%, 44% and 59% of genes from PS origin (Sophie Danvy, personal communication). Within the SF breed, the SF8 (not differentiated from the PS breed) was distinct from SFA97 and SFB98 populations. This result was in agreement with previous results from pedigree data [30]: the SF8 was found to have 98% of genes from PS origin. The three draught breeds ARD, AUX and TDN, were found to be quite similar, which is linked to a common historical and geographical origin (north of France) [27]. Iberic breeds (LUS and PRE) were also found to be genetically quite close. These results and the fact that according to Bayesian approaches, the likelihood became stable before *K *reached the number of breeds, indicate that the most relevant division is situated at a level superior to that of the breeds [31]. Such a subdivision of the whole set can be explained by the existing crossbreeding management system in several horse populations.

### Conservation priorities

In the present study, an almost comprehensive sampling of French breeds was achieved. The different approaches used gave an estimation of the contribution of each breed to the whole French horse stock. Petit [32] has proposed allelic richness as a good parameter to evaluate the genetic diversity of a population, useful as an indicator of past bottlenecks [33]. In our study, the POIT breed was found to have the lowest allelic richness and also one of the lowest within-breed contributions to diversity according to the two other methods used in the study. Because of the strong correlation with the mean number of alleles, the concept of allelic richness interest seemed to be of limited value in our study.

The results given by the aggregate diversity and gene diversity approaches were slightly correlated. By definition, breeds with low contributions to aggregate and total diversities should have related breeds in the data set. Thus, ARD, TDN, and AUX breeds, which were genetically highly related, illustrate quite well such a hypothesis.

According to the approaches of Ollivier and Foulley [33] and Cabalero and Toro [11], populations that contributed a lot to the total diversity were mostly non-endangered breeds (AR, PS, SF, TF). There were, however, some differences between the two methods when considering the eight breeds classified as endangered or endangered/maintained by the FAO (ARD, AUX, BOUL, LAND, MER, POIT, POT, TDN). Using the approach of Ollivier and Foulley [33], contributions to aggregate diversity *D *of BOUL, MER and POIT breeds were quite high, and taking into account population size, *CP *was the highest for BOUL, LAND and POIT breeds. Using the approach of Caballero and Toro [11], *GD*_*T *_decreased only when LAND and POT breeds were removed, and those two breeds plus the BOUL breed should have been kept to optimise *GD*_*T*_. The differences can be explained by the methods used in the two approaches, particularly considering the evaluation of the contributions to between-diversity. Using the approach of Caballero and Toro [11], some Weitzman criteria, such as the twin property [22], were not applied: for instance, assuming that two populations are genetically identical but very different from the whole set, removing one of them will largely decrease *GD*_*BS*_, which will not be the case when using the Weitzman approach. However, one advantage of the approach of Caballero and Toro [11] is the fact that there is no need to give weight to within- and between-diversities to compute total diversity, since by definition *GD*_*T *_is the sum of *GD*_*WS *_and *GD*_*BS*_. In fact, our results outline that both approaches should be considered as complementary to identify which breeds have to be taken into account in a context of genetic resource management. Therefore, conservation priorities should concern particularly BOUL, LAND, MER, POIT and POT breeds.

Another advantage of the method of Caballero and Toro [11] is the possibility of computing the contribution of each population to optimise total diversity. Such an approach was designed to conserve a large diversity of alleles. Therefore, it is not surprising to notice that the three breeds (PFS, SF, BOUL) that should have the highest contribution to optimise genetic diversity represent the three identified genetic differentiated groups. The importance of the PFS breed is due to the fact that this synthetic pony breed has the largest number of alleles. SF, another composite breed, has a smaller variability but carries alleles representative of the warm-blooded breed group, while the BOUL breed carries alleles seldom present in the two other breeds but frequent in draught horses.

Finally, several considerations have to be taken into account before taking final conservation decisions [34], such as the special range of performances for given traits, current production systems associated to the breed, socio-cultural value, or dynamics of the group of breeders. Between 1998 and 2003, births remained more or less stable for BOUL, LAND, POIT and POT breeds, but decreased for the MER breed [35]. In the endangered breeds, specific uses should be supported to maintain a demand for such horses (production of mules for the POIT breed, ecotourism for local breeds, draught activities, meat production). Genetic variability should also be managed, especially since some of these breeds constitute a pool of original genes (BOUL, MER and POIT) (see Figure 3). For instance, sires with different origins should be used [36]. When populations of the same breed are raised in other countries (such as the POT breed in Spain [31]), regular exchanges should be organised between both countries to maintain a relatively large variety of reproducers.

## Conclusion

Based on this study, horse breeds raised in France can be clustered into four groups. These groups were found to be meaningful according to the use of breeds, morphological characteristics and/or geographical origins. The combined use of different methods allowed us to identify breeds for which conservation efforts should be a priority, in order to preserve the maximum genetic variability. Since several horse studies have used similar panels of markers [7,9,10], it would be interesting to merge the corresponding data.

## Competing interests

The authors declare that they have no competing interests.

## Authors' contributions

JCM carried out the genotyping. AR contributed to the description of the populations and carried out the sampling collection. LC performed the preliminary analysis. GL carried out the computational analysis and prepared the manuscript. XR participated in the computational analysis and preparation of the manuscript. CDB participated in the preparation and the revision of the manuscript. EV participated in the design of the study and the revision of the manuscript. All authors read and approved the final manuscript.

## Supplementary Material

Additional file 1 **Supplementary Figure 1**. Neighbour-Net for the 34 horse breeds, based on Reynolds D_*R *_distance Click here for file

Additional file 2 **Supplementary Figure 2**. Correspondence analysis of allele frequencies for 33 of the populations studied (PRW is not included). The projection is shown on the first two axes. Click here for file

Additional file 3 **Supplementary Figure 3**. Evolution of mean ln of likelihood according to K on twenty runs (standard deviation indicated) Click here for file
