# Genetic diversity of a large set of horse breeds raised in France assessed by microsatellite polymorphism

**DOI:** 10.1186/1297-9686-41-31

**Published:** 2009-03-19

**Authors:** Grégoire Leroy, Lucille Callède, Etienne Verrier, Jean-Claude Mériaux, Anne Ricard, Coralie Danchin-Burge, Xavier Rognon

**Affiliations:** 1 AgroParisTech, UMR1236 Génétique et Diversité Animales, 16 rue Claude Bernard, F-75321 Paris, France; 2 INRA, UMR1236 Génétique et Diversité Animales, 78352 Jouy-en-Josas, France; 3 LABOGENA, F-78352 Jouy-en-Josas, France; 4 INRA, UR631 Station d'amélioration génétique des animaux, BP 52627, 31326 Castanet-Tolosan, France

## Abstract

After the recent publication of our article (Leroy, *Genetics Selection Evolution *2009 41:5), we found several errors in the published Table Three, concerning the computation of contribution to within-breed diversity (*CW*). We apologize to the readers for these errors, which are corrected in the present erratum.

## Correction

Table Three (see Table 1 of this erratum) of our recently published paper [1] contains several errors. Here we present the corrected version of Table Three (see Table 2 of this erratum) and explain the new data. The authors regret the errors.

**Table 1:** Original and incorrect Table Three presented in Leroy *et al. *(2009)

Breed code	Nb of breeding animals in 2005		Pr. extinction	Agregate diversity and cryopreservation potential (Ollivier and Foulley, 2005)				Loss or gain of diversity when a breed is removed and contributions to optimal diversity (Caballero and Toro, 2002)			
			
	Males	Females		*CW*	*CB*	*D*	*CP*	Δ*GD*_*WS*_	Δ*GD*_*BS*_	Δ*GD*_*T*_	*C*_*i*_
AA			0.11	0.35	0.85	0.39	0.10	-0.0013	-0.0018	-0.0031	0%
AR	480	2 130	0.03	0.29	10.90	1.25	0.35	-0.0015	-0.0010	-0.0026	0%
ARD	187	1 417	0.08	-0.48	1.33	-0.32	0.10	0.0031	0.0001	0.0032	0%
AUX	24	248	0.57	-0.19	3.14	0.11	1.79	0.0023	-0.0005	0.0018	0%
BOUL	58	540	0.24	-0.27	12.35	0.87	2.95	0.0040	-0.0023	0.0018	6%
BR	621	6 380	0.02	-0.38	5.57	0.16	0.12	0.0016	0.0009	0.0024	0%
CAM	118	837	0.12	0.00	7.99	0.73	0.97	-0.0018	0.0013	-0.0006	0%
COBND	63	760	0.21	-0.06	2.42	0.16	0.52	-0.0017	0.0019	0.0002	2%
COMT	856	7 073	0.02	-0.25	3.63	0.11	0.06	0.0000	0.0015	0.0015	0%
LAND	22	73	0.74	0.06	3.99	0.41	2.95	-0.0029	0.0016	-0.0014	2%
MER	93	1 012	0.15	-0.04	10.41	0.91	1.53	0.0000	0.0001	0.0001	0%
PER	183	2 461	0.07	-0.32	4.60	0.12	0.34	0.0006	0.0014	0.0020	0%
PFS	100	949	0.14	0.39	1.93	0.53	0.27	-0.0055	0.0024	-0.0031	70%
POIT	39	199	0.38	-0.43	12.60	0.75	4.83	0.0069	-0.0030	0.0039	0%
POT	94	910	0.15	0.19	1.33	0.29	0.20	-0.0040	0.0024	-0.0016	5%
PS	369	8 049	0.04	0.50	6.17	1.02	0.22	-0.0001	-0.0041	-0.0042	1%
SF	474	11 700	0.03	0.45	1.33	0.53	0.04	-0.0024	-0.0013	-0.0037	15%
TDN	16	183	0.85	-0.17	1.93	0.02	1.64	0.0032	-0.0009	0.0022	0%
TF	527	15 950	0.02	0.36	7.51	1.01	0.18	-0.0002	-0.0029	-0.0032	0%

Sum				0	100	9.054		0	-0.043	0.043	100%

*CW *= contribution to within-breed diversity; *CB *= contribution to between-breed diversity; *D *= aggregate diversity;*CP *= cryopreservation potential; Δ*GD*_*WS *_= loss or gain of gene diversity within populations when breed is removed; Δ*GD*_*BS *_= loss or gain of gene diversity between populations when breed is removed; Δ*GD*_*T *_= loss or gain of total diversity when the breed is removed; *C*_*i *_= contribution of the breed to optimise *GD*_*T*_

**Table 2:** Corrected Table Three

Breed code	Nb of breeding animals in 2005		Pr. Extinction	Agregate diversity and cryopreservation potential (Ollivier and Foulley, 2005)				Loss or gain of diversity when a breed is removed and contributions to optimal diversity (Caballero and Toro, 2002)			
			
	Males	Females		*CW*	*CB*	*D*	*CP*	Δ*GD*_*WS*_	Δ*GD*_*BS*_	Δ*GD*_*T*_	*C*_*i*_
AA	119	1 443	0.11	0.18	0.85	0.24	0.10	-0.0013	-0.0018	-0.0031	0%
AR	480	2 130	0.03	0.21	10.90	1.18	0.35	-0.0015	-0.0010	-0.0026	0%
ARD	187	1 417	0.08	-0.46	1.33	-0.30	0.10	0.0031	0.0001	0.0032	0%
AUX	24	248	0.57	-0.32	3.14	-0.01	1.79	0.0023	-0.0005	0.0018	0%
BOUL	58	540	0.24	-0.60	12.35	0.57	2.95	0.0040	-0.0023	0.0018	6%
BR	621	6 380	0.02	-0.24	5.57	0.29	0.12	0.0016	0.0009	0.0024	0%
CAM	118	837	0.12	0.27	7.99	0.97	0.97	-0.0018	0.0013	-0.0006	0%
COBND	63	760	0.21	0.24	2.42	0.44	0.52	-0.0017	0.0019	0.0002	2%
COMT	856	7 073	0.02	-0.01	3.63	0.32	0.06	0.0000	0.0015	0.0015	0%
LAND	22	73	0.74	0.48	3.99	0.79	2.95	-0.0029	0.0016	-0.0014	2%
MER	93	1 012	0.15	0.02	10.41	0.96	1.53	0.0000	0.0001	0.0001	0%
PER	183	2 461	0.07	-0.10	4.60	0.33	0.34	0.0006	0.0014	0.0020	0%
PFS	100	949	0.14	0.78	1.93	0.89	0.27	-0.0055	0.0024	-0.0031	70%
POIT	39	199	0.38	-1.00	12.60	0.23	4.83	0.0069	-0.0030	0.0039	0%
POT	94	910	0.15	0.58	1.33	0.64	0.20	-0.0040	0.0024	-0.0016	5%
PS	369	8 049	0.04	0.01	6.17	0.57	0.22	-0.0001	-0.0041	-0.0042	1%
SF	474	11 700	0.03	0.34	1.33	0.43	0.04	-0.0024	-0.0013	-0.0037	15%
TDN	16	183	0.85	-0.41	1.93	-0.20	1.64	0.0032	-0.0009	0.0022	0%
TF	527	15 950	0.02	0.02	7.51	0.70	0.18	-0.0002	-0.0029	-0.0032	0%

Sum				0	100	9.054		0	-0.043	0.043	100%

*CW *= contribution to within-breed diversity; *CB *= contribution to between-breed diversity; *D *= aggregate diversity;*CP *= cryopreservation potential; Δ*GD*_*WS *_= loss or gain of gene diversity within populations when breed is removed; Δ*GD*_*BS *_= loss or gain of gene diversity between populations when breed is removed; Δ*GD*_*T *_= loss or gain of total diversity when the breed is removed; *C*_*i *_= contribution of the breed to optimise *GD*_*T*_

## Results

### Partition of diversity

Errors concern the computation of the *CW *component developed by Ollivier and Foulley [2]. In the new version, *CW *ranged from -1 to 0.78. As aggregate diversity *D *is defined as a linear combination of *CW *and contribution to between-breed diversity, column *D *had also to be corrected, and ranged from -0.30 to 1.18. Consequently, the Pearson correlation between *CW *and Δ*GD*_*WS *_was found to be -1 (instead of -0.72 in the previous version), and the Pearson correlation between *D *and Δ*GD*_*T *_was found to be -0.59 (*P *= 0.008).

## Discussion

### Conservation priorities

In spite of the above modifications, the populations that contributed most to the total diversity, according to the approaches of Ollivier and Foulley [2] and Caballero and Toro [3], still remain mostly the non-endangered breeds (AR, PFS, TF) [instead of AR, PS, SF, TF in the previous version].

On the contrary, when considering the eight breeds classified as endangered or endangered/maintained by the FAO (ARD, AUX, BOUL, LAND, MER, POIT, POT, TDN) and the approach of Ollivier and Foulley [2], a change is noted for the breeds exhibiting the highest contributions to aggregate diversity *D*, which are now MER, LAND and POT, instead of BOUL, MER and POIT.

Finally, since the discussion on breed conservation is based on the use of several other methods and parameters, the above new results do not change our recommendations on which breeds specifically need support.
