# Supplementary figures and images for: Genetic diversity of a large set of horse breeds raised in France assessed by microsatellite polymorphism
Source: Genet Sel Evol. 2009 Jan 5;41(1):5. doi: 10.1186/1297-9686-41-5 (PMC3225878; doi:10.1186/1297-9686-41-5)

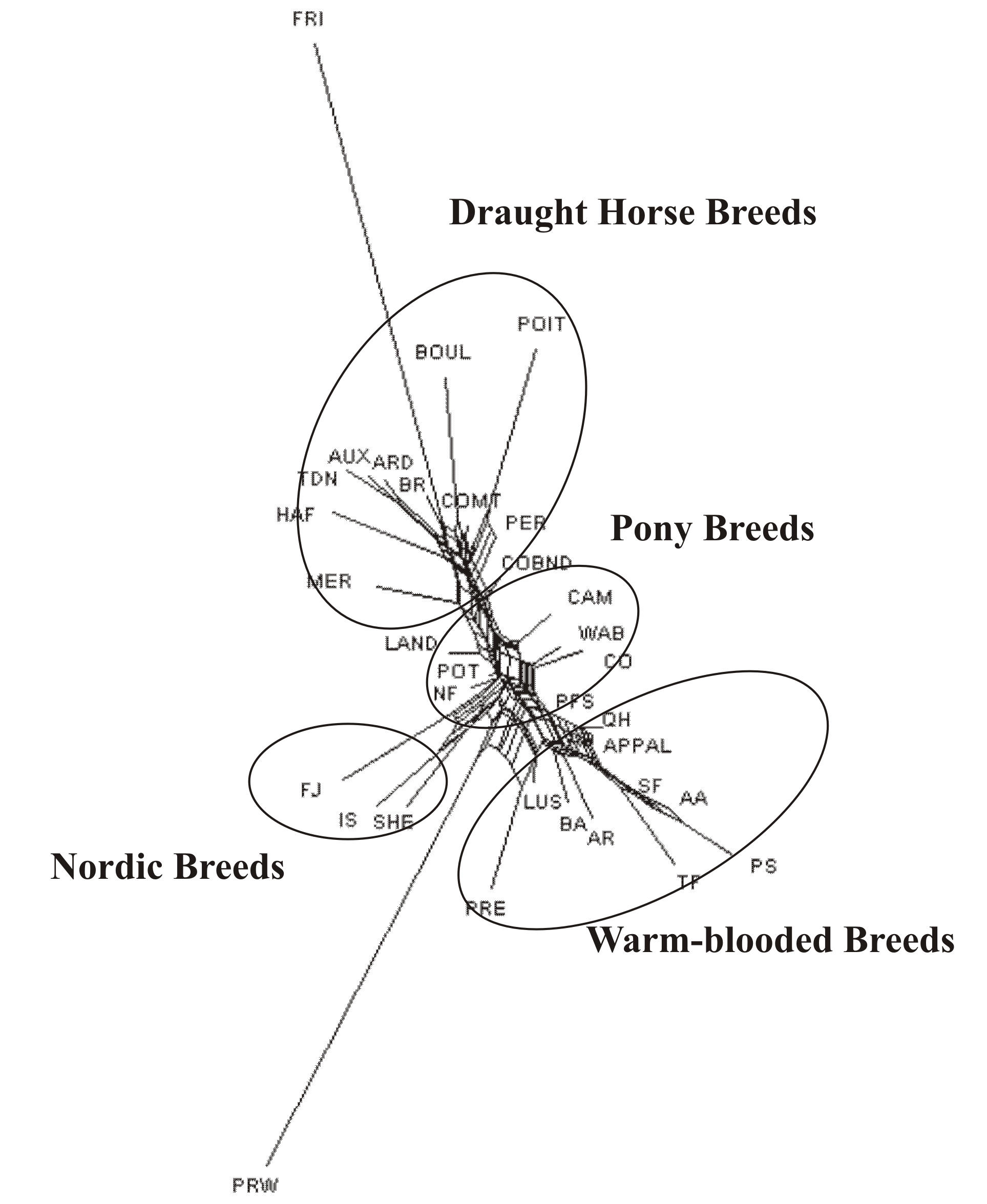

Supplement: Additional file 1 — Supplementary Figure 1. Neighbour-Net for the 34 horse breeds, based on Reynolds DR distance [file 1297-9686-41-5-S1.tiff]

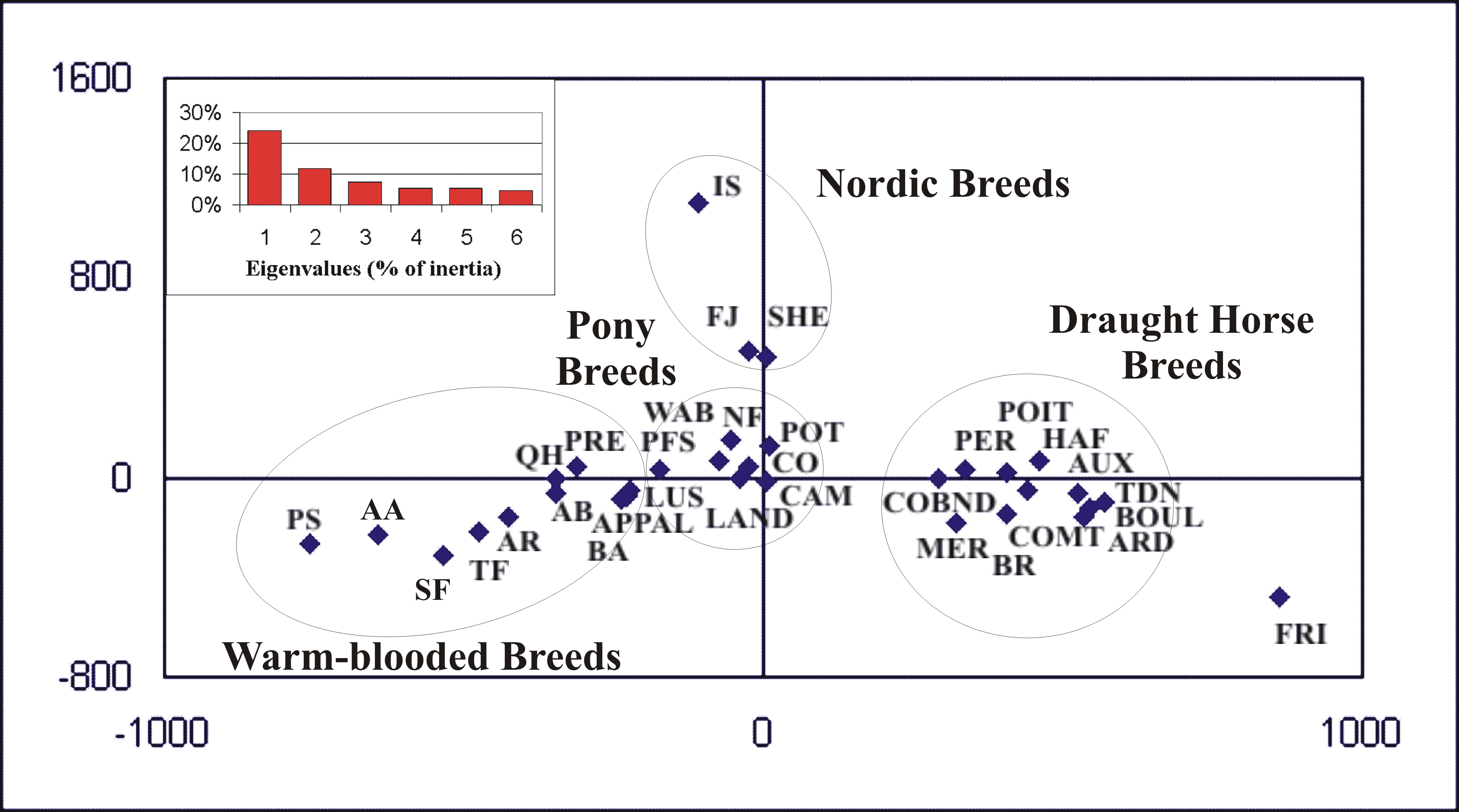

Supplement: Additional file 2 — Supplementary Figure 2. Correspondence analysis of allele frequencies for 33 of the populations studied (PRW is not included). The projection is shown on the first two axes. [file 1297-9686-41-5-S2.tiff]

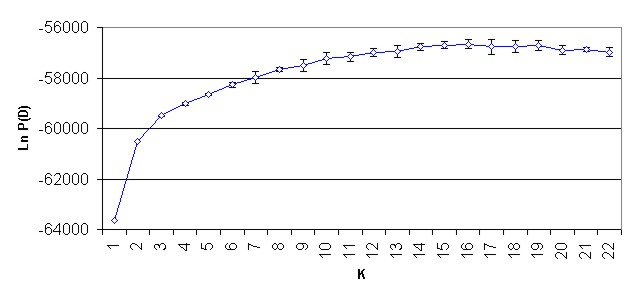

Supplement: Additional file 3 — Supplementary Figure 3. Evolution of mean ln of likelihood according to K on twenty runs (standard deviation indicated) [file 1297-9686-41-5-S3.tiff]
